# Navigating Dementia: Political Materialities of Public Transport in the All‐Ageing Metropolis

**DOI:** 10.1111/1467-9566.70017

**Published:** 2025-03-09

**Authors:** James Rupert Fletcher

**Affiliations:** ^1^ Information, Decisions and Operations Division School of Management University of Bath Bath UK

**Keywords:** Alzheimer, disability, infrastructure, mapping, mobility, new materialism

## Abstract

Age‐ and dementia‐friendliness are major areas of contemporary urban policy and scholarship, seeking to maintain older people and those with cognitive impairment in their own homes and communities. Such work relies on architectural augmentation to maximise the functionality of ageing bodies and minds and has been criticised for conceptualising place as a static unidirectional determinant of individual disability. New materialist scholarship on place, ageing and disability is challenging such conceptions, theorising people and places as dynamically co‐constituting socio‐material ecologies that co‐age and co‐dis/enable. Moreover, a critical tradition is resituating dementia‐friendliness in the materialising capacities of political economy in everyday public living with dementia. Building on that work, this paper reports on a yearlong creative go‐along ethnography conducted with eight passengers with dementia on public transport in Greater Manchester, UK. Through interviewing, multimedia generation and map‐making, their stories highlight unequal materialisations of (im)mobilities in relation to the fractious political economies of urban infrastructures in the all‐ageing metropolis.

## 1 Cost‐Friendly Stasis

‘Ageing‐in‐place’ and ‘age‐friendly communities’ represent major global policy platforms dedicated to supporting older people to live in their own homes and communities for as long as possible (Webber, May, and Lewis 2023). Corresponding strategies span the provision of home adaptation (e.g., handrails), neighbourhood accessibility enhancement (e.g. public seating) and community initiatives to strengthen social infrastructures (e.g., resident activities) (Hammond and Rodgers 2023). ‘Dementia‐friendly communities’ are inspired by age‐friendliness, manifesting a comparable emphasis on maintaining independence in place—this time for people with dementia rather than older people per se—through interventions such as signage, lighting and community training (Hebert and Scales 2019).

As global ageing emerged as a prominent political economic concern through the late 20th century, academic attention began to focus on place‐based approaches to different trajectories of ageing and particularly experiences of later life disability (Pain, Mowl and Talbot 2000). Politically, age‐friendly and ageing‐in‐place strategies have long been critiqued for foregrounding individual independence and resilience as the key to remaining in place as one ages (Dalmer 2019). These approaches institute conservative moral and fiscal rationales for limiting welfare provision and emphasising personal responsibility, responding to late‐20th century demographic alarmism portending that population ageing would economically imperil states by increasing care expenditure and reducing productivity. Dementia‐friendly initiatives echo these shortcomings and have hence been criticised for facilitating welfare removal and the corresponding responsibilisation of individuals and families (Fletcher 2024a; R. Ward et al. 2022a).

Conceptually, critiques has challenged underwhelmingly static and unidirectional operationalisations of place and its relations with later life disability that have typified the friendliness movements (Urban 2021). Manifesting a traditional *determinants of health* perspective, age‐friendly and dementia‐friendly agendas historically depicted places as fixed settings within which individuals experience intrinsic and universal degeneration. For example, advocating age‐friendly environmental adaptation, WHO (2024) states: ‘Environments play an important role in determining our physical and mental capacity across a person's life course and into older age and also how well we adjust to loss of function’. Similarly, in a recent state of the science review, Hebert and Scales (2019: 1881) found that 75% of published dementia‐friendly scholarship focussed on ‘environmental adaptation to enhance the physical, social, and functional ability of PWD’. In response, global policy and associated scholarships approached places as determinants of disability that could be architecturally augmented to maximise the individual's functional ability to remain there despite their ageing bodies and minds (Buffel and Phillipson 2023).

Critical ageing scholars are questioning characterisations of intrinsic personal decline in static places. Adapting long‐standing arguments from human geography regarding the dynamism of place, social gerontologists are emphasising that places and people co‐age, comprising ageing material entanglements that change together through time (Höppner and Urban 2019). This work is also extending to dementia. For instance, Brittain and Degnen (2022, 429) advocate ‘imagining dementia friendly communities as a complex intersection where care both makes—and is made by—social and material relations’. Ward and colleagues (2022a) caution that positively framed neighbourhood‐friendliness programmes can obscure the pathological influence of political economic forces, such as austerity politics and welfare retraction, and the everyday precarities that they can generate for people with dementia. In this paper, I extend this critical work on political materialities of ageing, dementia and place by analysing materialisations of urban polity in the everyday lives of passengers with dementia using public transport.

## 2 Toward All‐Ageing Disabilities

Critique of conceptualisations of place vis‐à‐vis later life and associated disability has been animated by the wider (new) material turn (Urban 2021). Scholars have sought to challenge material/symbolic binaries that have historically typified social science (classically in historical materialist vs. cultural turn debates (Fox and Powell 2021)). They have instead theorised universes made up of more‐than‐human matter, both material and meaningful, which has relational agentic capacities for mattering, co‐constituting material and meaningful social worlds (Fletcher 2024b; Andrews, Griffin, and Phoenix 2024; Boyer and Spinney 2016). The long‐standing gerontological theorisation of age and ageing as sociopolitical locations, rather than simply personal physiological characteristics, has latterly been enlivened by the new materialist turn, albeit most notably in relation to technology (Gallistl et al. 2024; Höppner and Urban 2019). This represents an exciting moment in social gerontological thought and the sociology of ageing, which has long self‐bemoaned its need for theoretical revitalisation (Kohli 1988; Higgs 2023).

Andrews (2022) summarises this new materialist interest in ageing as attending to ‘all‐ageing’ ‘under the material press of time’. He notes that the common policy and scholarship refrain ‘the world is ageing’ is unwittingly astute because the entire world and all its sociomaterial facets are indeed co‐ageing. Recognising all‐ageing, new materialist scholars are conceptualising ageing as a multidirectional, sociomaterial and relational process of co‐change through time (Gallistl et al. 2024; Höppner and Urban 2019). New materialism's material sensibilities resonate with mainstream recognition of place as deeply influential in ageing processes. However, rejecting the traditional unidirectionality of ‘friendly’ scholarship, new materialism offers a symbiotic view of places, peoples, plants, animals and technologies as co‐constituents of ageing macrocosms with multiple capacities to enable and disable us under time's material press (Andrews and Duff 2022). Although celebrating these contemporary trajectories toward a new materialist co‐ageing‐with‐places, Andrews and Read (2024) have recently critiqued the tradition for remaining too tied to the human as *the* ageing subject. This paper takes up that critique by centring place and politics as ageing subjects.

At the same time, we must be wary of this ‘*post*humanist’ [emphasis added] drive for ‘even flatter ontology’ leading toward overly *anti*humanist or politically naive sensibilities (Andrews and Read 2024). New materialism has been critiqued for depoliticising its study phenomena and obscuring political economies of later life disability, owing to its redistribution of agencies away from humans and the consequent dilution of power (Fletcher 2024b; Choat 2018). However, scholars have responded with decidedly critical work, emphasising experiences of ageing, disability and injustice (e.g., Changfoot and Rice 2020; Manchester and Jarke 2022). For instance, Manchester and Jarke (2022) have advocated making visible the sociomaterialities of life with dementia in care settings, co‐creating methods to generate new connections and resist oppressive ecologies that can isolate people as passive singular recipients of material assistive interventions. Offering a rich account of cognitive impairment lived through a tumultuous political economy, this paper contributes to the burgeoning new materialist tradition of explicating injustices that intensify later life disability.

Among this disability, perhaps none is so confounding as that involving dementia. Dementia is strongly associated with older age, both epidemiologically and symbolically, and has long operated as a feared manifestation of later life disability (Higgs and Gilleard 2016). The nature of dementia as a mode of ‘cognitive’ impairment has troubled the social theorisation of disability, which has focussed on more intuitively physical disabilities (famously exemplified by a wheelchair user prevented from voting by step‐only polling station access), leaving dementia conceptually orphaned (Shakespeare, Zeilig, and Mittler. 2019). Among its many promises and challenges, a new materialist account of ageing offers exciting prospects for engaging with cognitive impairment as an important constituent of later life disability within material ecologies (Fletcher 2023a), emphasising an attentiveness to the mesh of neuropsychological and sociopolitical materialities at stake in dementia (Fletcher 2023b). Work in this vein is emphasising the centrality of diverse sociomaterialities in co‐constituting everyday experiences of dementia in public spaces in relation to broader political and economic forces (Brittain and Degnen 2022; R. Ward et al. 2022a). In particular, the work of Ward and colleagues is challenging traditional *determinant* approaches to dementia and place by exemplifying how diverse modes of living with dementia happen with and through dynamic localities (R. Ward et al. 2018, 2022a). In line with their work, this paper further troubles categorical, static and interventionist approaches to dementia‐friendly places.

## 3 Moving With Manchester

The paper reports findings from the Wellcome Trust [grant: 222193/Z/20/Z] funded IN‐CITU project, a yearlong study of the experiences of passengers with dementia as they used public transport across Greater Manchester, a ceremonial county in northwest England and the UK's second most populous urban area (∼3,000,000). Greater Manchester and Manchester were the UK's first age‐friendly metropolitan region and city, and the local government has long emphasised age‐friendly policy with dementia‐friendly initiatives overseen by a dedicated government body, Dementia United. This focus extends to friendly transport strategies for the region's bus, tram and rail networks. Transport is one of the most significant challenges faced by people with dementia, for whom driving licence revocation can lead to immobilisation and isolation (Fletcher 2024c). Greater Manchester's pursuit of friendly transport is coinciding with a major re‐municipalisation programme, beginning in 2023, the first of its kind in the UK following decades of privatisation. Transport re‐municipalisation contrasts Greater Manchester's broader political economic trajectory, with recent urban redevelopment criticised for exacerbating inequalities and serving international capital flows at the expense of residents.

Following 2008's global recession, national austerity politics decimated local authority budgets with 40% decreases between 2010 and 2020 (Atkins and Hoddinott 2023). In response, Manchester became a posterchild for devolved financial innovation. In 2014, the council sold £1 billion of land to the Abu Dhabi United Group for the ‘Manchester Life’ luxury flat development, a move lauded by the national government as exemplifying financial competence (HM Treasury 2014). However, attempts to enhance the city's financial attractiveness led the council to sell land cheaply and facilitate offshoring. Manchester Life's subsidiary paid just £10,000 tax on £26 million revenues (Goulding, Lever & Silver 2022). This investor‐friendliness is emblematic of Manchester's political materialisation as a model for 21st century financialised regeneration amidst interlocking global and national pressures (Rose 2024). With cheap land, densified young professionals, and 20% annual rent increases, Manchester now offers the UK's highest rental yields (Aldermore 2023). Its financial promise relies on imageries of affluent young renters in inner‐city skyscrapers.

In this context, *IN‐CITU* sought to better understand cognitive disability in urban ecologies by accompanying passengers with dementia as they used Greater Manchester's public transport to make a regular journey. Throughout 2023, I accompanied eight passengers (Table 1) on 17 journeys, covering 131 miles in 17 h of travel. Participants had formal dementia diagnoses, lived in Greater Manchester and regularly used public transport. They were recruited with the assistance of Dementia United (*n* = 2), the Greater Manchester Mental Health NHS Foundation Trust (*n* = 4) and word of mouth (*n* = 2). Project advertisements were handed to potential participants by the listed organisations, and with their consent the contact information of interested parties was given to me. I assessed capacity to consent during preliminary meetings at participants' homes, a legal requirement for conducting research with people with cognitive impairments in England. Procedural ethical approval was granted by the NHS North West ‐ Greater Manchester South Research Ethics Committee [reference: 22/NW/0142].

**TABLE 1:** Participants.

Pseudonym	Age	Diagnosis	Borough	Journeys	Mode	Home	Co‐residents	Profession^a^
Paul	63	Vascular dementia	Bolton	1	Bus	Terrace	Wife, 2 dogs	Administrator
Barbara	71	Alzheimer's disease	Salford	6^b^	Bus/tram	Semi	Granddaughter	Nurse
Judy	82	Alzheimer's disease	Salford	2^b^	Bus/tram	Bungalow	Husband	Driver
Stanley	86	Alzheimer's disease	Manchester	2	Bus/tram	Detached	Wife	Solicitor
Marilyn	80	Vascular dementia	Rochdale	1	Bus	Semi	Husband	None
Liz	75	Mixed dementia	Bury	1	Tram	Semi	None	Administrator
Joyce	76	Vascular dementia	Manchester	5	Bus	Flat	Cat	Dog trainer
Nicole	80	Alzheimer's disease	Stockport	1	Bus	Flat	None	None

^a^ Prior to retirement.

^b^ Both Judy's journeys included Barbara, Paul travelled with his wife, Marilyn travelled with her husband, all others travelled alone.

The project implemented a creative go‐along ethnography, accompanying participants on their usual journeys and using a range of methods to document the trips, including field notes, interviewing, audio recording, photography, videography and geolocating. Creative ethnography is a positive dementia research methodology because of its adaptability to participant needs and preferences, its capacity for joyfully engaging people who may be wary of participation and its rejection of conventional language‐based question–response methods that can be challenging for people with cognitive impairments (Hogger, Fudge, and Swinglehurst 2023). Participants were first interviewed in their homes about their public transport experiences and trips were arranged during the meeting. Participants were then accompanied on their typical journeys using any mode of public transport. These journeys were geolocated using the Komoot app on an iPhone 14 Pro, and the geolocation data were visualised using the GPX.studio platform. Picomic 2 cordless lapel microphones recorded our conversations, which I later transcribed and the recorded soundscapes were segmented using the Audacity audio editor. Participants were invited to capture photographs and video using the iPhone, though some preferred to direct me to document things.

Each journey produced a rich dataset of geolocation (.gpx), geolocation visualisation (.png), audio (.mp3), photographs (.jpg), videos (.mp4), transcript and field notes (.doc). The data were collated into a GeoDoc using ATLAS.ti analysis software. GeoDocs use the open‐source Open Street Maps format as an editable world map that can be built up with multimedia. I used the ‘coding’ function to pin media (uploaded to ATLAS.ti as separate ‘documents’) to the map, following the geolocation route and used the ‘quotation’ feature to insert transcript and note segments. Each item added to the map was classified according to a project key, implementing unique identifiers to capture the interrelatedness of different data forms (Table 2). All media and all codes relating to a specific journey were grouped and colour‐coded in ATLAS.ti. The final interactive journey map encompassed 270 documents, 684 codes and 922 quotations.

**TABLE 2:** Key.

Identifier				Description
P01				Participant 1
	P01j001			Participant 1 journey 1
		P01j001k		Participant 1 journey 1 gpx file
		P01j001g		Participant 1 journey 1 gpx visualisation
		P01j001a		Participant 1 journey 1 complete audio
			P01j001a001	Participant 1 journey 1 audio clip 1
		P01j001t		Participant 1 journey 1 complete transcript
			P01j001a001t	Participant 1 journey 1 audio clip 1 transcript
		P01j001n		Participant 1 journey 1 notes
			P01j001p001	Participant 1 journey 1 photograph 1
			P01j001v001	Participant 1 journey 1 video 1

My map‐making approach to the dataset, which I characterise as ‘journeying analysis’, was exploratory and refined throughout the project. I began with ATLAS.ti's basic capabilities and experimented, using them in different ways until I developed an approach that satisfied my purposes. Namely, I required an analysis approach that allowed me to synthesise all data forms, from geolocations to soundscapes, in a coherent and intuitive format that captured their place, motion and connection. Digital tools for aggregating multimedia were key here as conventional cartographic documentation of social life have been criticised for conjuring static fields of power (R. Ward et al. 2022a). This experimentation was a form of creative methodology, exemplifying the under‐explicated potentials for familiar software to furnish creative sociologies, particularly when conducting data analysis.

The core praxis of journeying analysis is the act of map‐making, compiling a heterogeneous collection of data forms into something with a journey‐like quality. Map‐making is an analogue of the general meaning‐making processes that typify data analysis more broadly. Indeed, cartography has a rich history as a means of social scientific data analysis and is suited to examinations of spatial and mobility experiences. The act of map‐making sensitises the analyst to the relations between and within spaces and produces comparative visualisations of overlapping and intersecting—hence connected—data. A famous example of such a connective mapping approach to everyday lives was John Snow's 1854 cholera map. Digital map‐making based on geolocation data can also engage with and blend time (and by extension motion, i.e., place × time) into the analytic process by retracing an interactive version of the journey place by place.

This inclination toward time, movement and action allows a journeying analysis to sensitise the analyst toward certain types of meaning making, but does not impose prerequisite interpretations and hence facilitates creative analysis. This methodological approach centres the possibility of data in line with the ‘nonrepresentational’ tradition (Andrews 2014). Nonrepresentational work seeks to produce possible (as opposed to authoritative) analytic accounts of a research problem that blur boundaries between researcher and reader, creating artful heuristics that different audiences can engage when trying to make sense of related phenomena (Bergmann 2016). This approach is particularly resonant with dementia research because it lends itself toward plurality of communication rather than seeking singular correct language‐based answers (M. Ward 2021). Manifesting these intellectual objectives, digital map‐making can offer useful strategies for invigorating critical new materialist understandings of ageing, disability and place. Although not the focus of this paper, I expand on journeying analysis at length elsewhere (Fletcher 2025).

Below, I present four participant stories that shed light on experiences of later life cognitive disability as phenomena emanating in political materialities.

## 4 Paul: Alienating Inequalities

**FIGURE 1:**
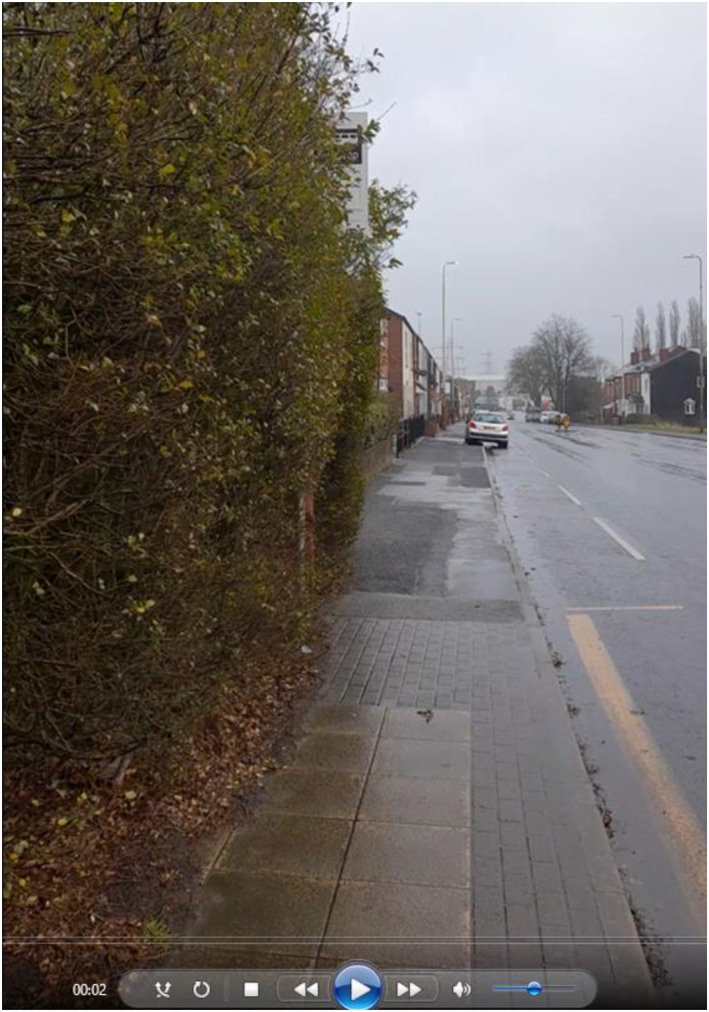
Video still of Paul's stop.

IN‐CITU began data generation on a cold January morning. I met Paul at his house in the borough of Bolton, in a 10‐mile hinterland of red brick terraces between Manchester city centre and Bolton town centre. Paul and his wife both had bad backs, but still wanted to catch the bus from the top of their street into central Manchester to visit a favourite café. Paul was relatively well served by the bus network, with a stop a few hundred yards from his door and a bus every 10 min. Unfortunately, that stop was merely a metal pole with a sign attached, erected at the top of a steep hill and hidden in a hedge (Figure 1). Paul struggled up the hill with mobility and breathing issues, then leant on the icy pole to catch his breath. The local church had fundraised to install seating but had only managed one bench the other side of the road. When the bus collected us, Paul and I sat side‐by‐side in the accessible seating at the front. We headed down the main road towards central Manchester.

**FIGURE 2:**
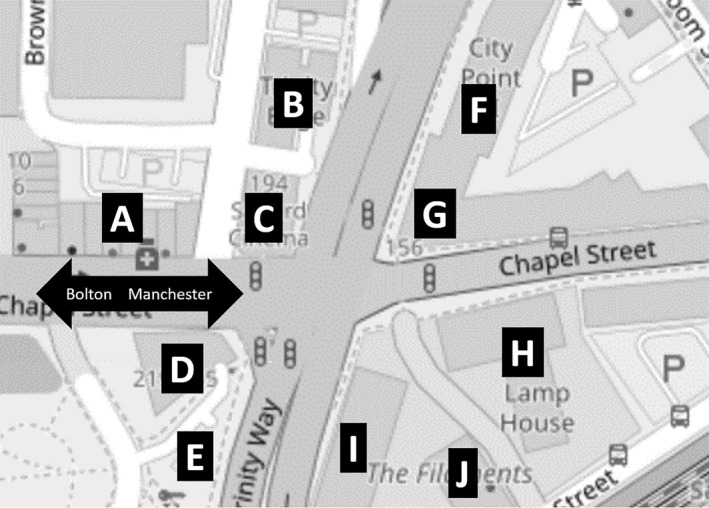
Storeys: outside ring‐road: A‐3, B‐4, C‐2, D‐4, E‐1; inside ring‐road: F‐7, G‐8, H‐21, I‐12, J‐16.

Central Manchester has the UK's largest number of high‐rises outside of London. Its recent construction boom has intensified the contrast between a towering centre and the two‐ to five‐storey buildings encircling it. Indeed, the city is renowned for a laissez‐faire approach to planning, lacking any formal building height restrictions, and for the concentration of development in its centre. This development entails perpetual walkway and road closures for resurfacing and redirection, so that the city, and one's ability to move through it, is materially fluid. Indeed, as we had embarked the bus, Paul's wife mentioned that we would need to disembark at a new stop due to road closures. Approaching Manchester from the west via the Chapel Street bus corridor, we crossed the ring road, which forms a boundary between the frenetic centre and its more mundane surrounds, demarcating entry into a palpably different kind of place (Figure 2).

At this literal and symbolic crossroads, replete with temporary traffic lights to accommodate the construction of a new pavement, Paul noted the challenges presented by Manchester's continual re‐materialisation:

Paul This gets problematic from here because it's, erm, they're always changing the route and I start thinking, ‘Where am I going to get off?’

Me So if you're going through the city centre, do you usually recognise most places?

Paul Erm, they're changing that many places, you don't recognise them

Paul articulated a problem shared by several participants. The constant transformation of central Manchester made it difficult to navigate, stripping out the material familiarities and routines that can be integral to supporting a normal life with dementia and even integral to grounding a sense of self (Varley 2008). Paul was born in Manchester. He was a ‘local’. However, this nominally lifelong relationship with places was effectively a series of relationships with places that only existed for periods of time. Rather than a relationship with Manchester's space, he had a relationship with Manchester's spacetime, a far more ephemeral entity with which to relate. This spacetime was made potent through encounters with buildings and roads, so that Paul was ultimately relating to ‘spacetimematterings’ in the moment (Gallistl and Wanka 2023), with the shifting columns of concrete and glass materialising the grander transformation, and hence ageing, of Greater Manchester as a whole.

Paul's wayfinding depended on spacetimematter, and his cognitive impairment undermined his abilities to adapt to Manchester's fast‐mutating spacetimematterings. Those mutations were driven by a torrent of local, national and global political economic forces. Paul's wayfinding difficulties emanated at the intersections of international property investment, liberal regional planning and cognitive impairment. This is a confluence to which I will return, but for now it is worth considering the next part of my journey with Paul. Our conversation continued, and given the difficulties that Manchester posed, I wondered whether he instead preferred travelling into Bolton town centre:

Me Do you ever go the other way? Do you go into Bolton?

Paul There's nothing there anymore. It's like one of these things where it's a ghost town now. All the major shops have closed down. All you've got left is, like, the pound shop and things like that

**FIGURE 3:**
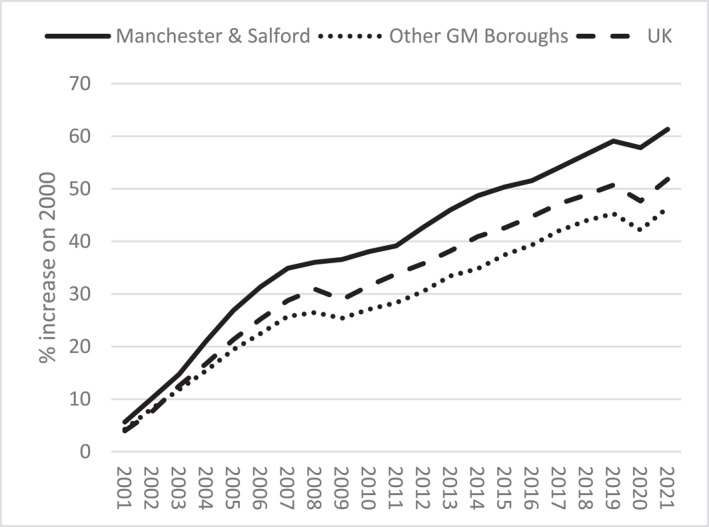
GDP growth (ONS 2023).

**FIGURE 4:**
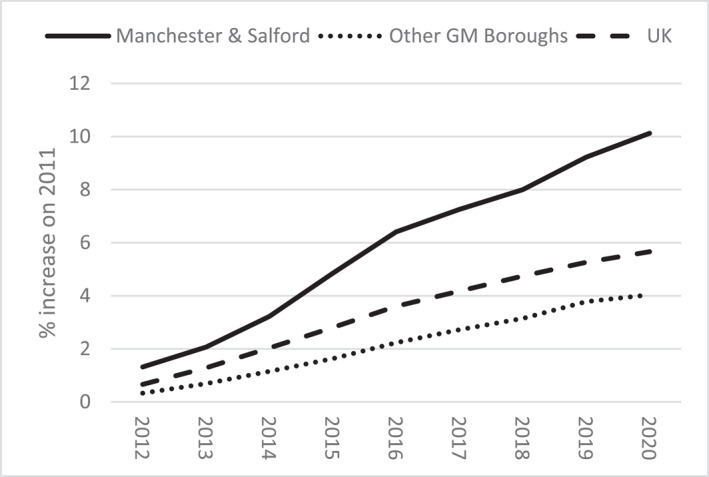
Population growth (ONS 2022).

**FIGURE 5:**
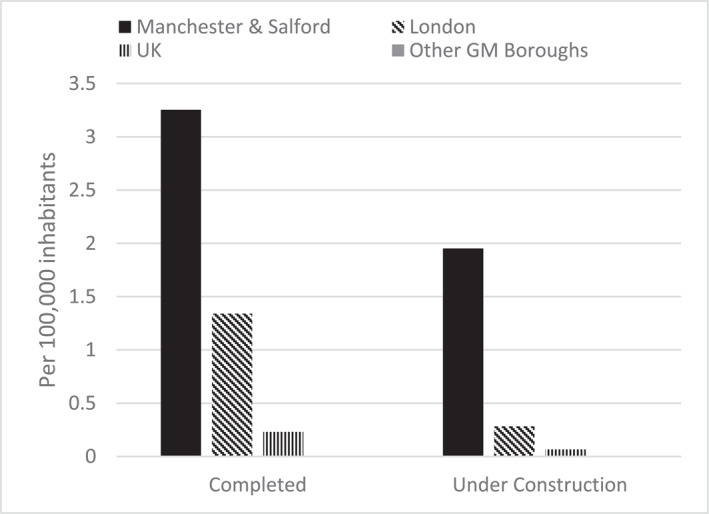
Developments 100m+, December 2023 (CTBUH n.d.).

Paul's reflections are indicative of inter‐borough inequalities that characterise Greater Manchester. The region's contemporary economic miracle is highly centralised in inner‐city Manchester and adjacent areas of Salford, whereas Greater Manchester's other boroughs track below UK averages across various metrics (Figures 3, 4, 5). Moreover, these inequalities are growing as inner‐city development compounds rather than trickling out. Over time, Greater Manchester's unequal political economy has materialised corresponding spatial inequalities, generating Paul's ‘ghost town’ of Bolton, which is only 10 miles from one of Europe's fastest developing cities. The resulting places are characterised by corresponding forms of alienation, pushing people out through too little activity or too much, comparatively exaggerating one another's disabling capacities.

Several participants felt similarly alienated by both Manchester's vibrant centre—difficult to navigate and not intended for them (i.e., more cashless businesses and expensive coffee shops)—and the struggling towns surrounding it—offering diminishing incentives to travel (i.e., closing shops and decrepit infrastructure) and generating depressing affects. To this end, both Liz and Nicole separately used the word ‘sad’ to describe their respective outlying highstreets in Blackley and Edgeley. I argue that such accounts indicate a political materialisation of alienation through the architectural‐affective co‐intensification of existing socioeconomic inequalities, which is made potently tangible by transport because passengers encounter stark sociomaterial differences as they move between places. Hence, the nature of movement provokes a unique sensory kinaesthetic experience of inequality (Spinney 2006). It is, however, important to recognise that alien‐city/sad‐town sentiment was not universal. Stanley felt at home in central Manchester, whereas Marilyn and Joyce enjoyed their respective town centres of Middleton and Wythenshawe, despite both recognising and regretting their decline. I will return to the tumultuous city centre, but next I want to focus on those outlying towns via Joyce's story.

## 5 Joyce: Segregated De‐Conditioning

**FIGURE 6:**
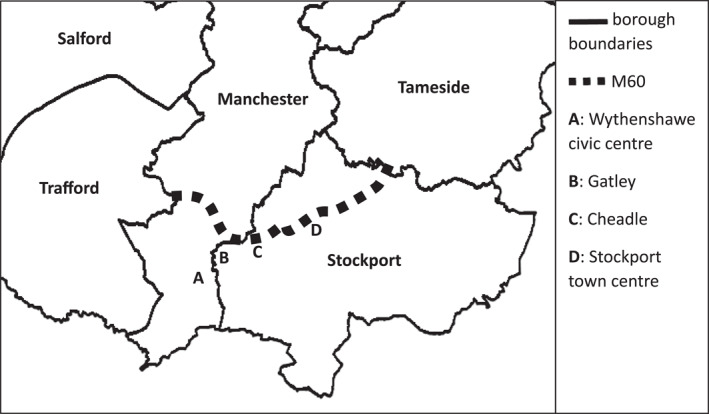
Southeast Greater Manchester.

Regional inequalities can generate feelings of Manchester's otherness from outlying communities, a sentiment echoed infrastructurally. Travelling south from the city centre to meet Joyce, I encountered this infrastructural otherness when crossing the motorway into Wythenshawe. Although only separated by 60ft of tarmac, Wythenshawe felt far removed from the city centre and affluent suburbs I had just traversed. Wythenshawe is characterised by mass homogenous housing. It rapidly expanded in the early‐20th century, becoming the UK's largest council estate, infamously devoid of amenities. It remains cut‐off, architecturally and affectively, from the rest of Manchester by the M60. Joyce felt greater affinity with Stockport than Manchester, having not ventured north of the M60 for years. Besides Wythenshawe civic centre, our journeys took us to Stockport town centre, Gatley and Cheadle (Stockport suburbs). Joyce's bus routes traced the motorway as her territorial boundary (Figure 6).

**FIGURE 7:**
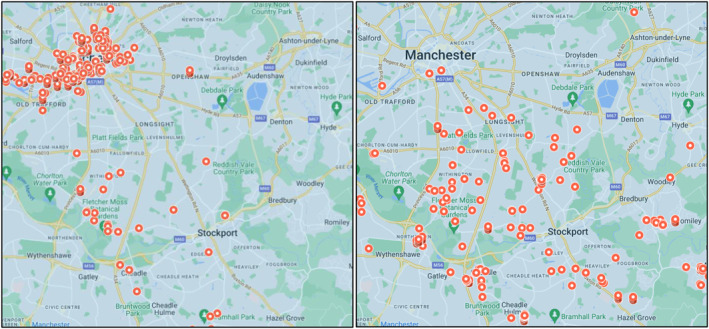
Left: flats for sale; right: retirement homes for sale (rightmove.co.uk search 29.01.2024).

I met Joyce at her retirement flat, which is one of many local developments catering to older residents. These developments exemplify how the spatial terrains of inequality articulated by Paul entail demographic resonances, including unequal population ageing. Between 2011 and 2021, 2/3 of Manchester's total population growth was made up of people aged under 65. Conversely, 2/3 of Stockport's growth was made up of people aged 65+. Today, although Manchester's population is unusually youthful, with a dependency ratio^1^ of 8.0, adjacent Stockport (3.3) is older than the UK average (4.3) (GMCA 2023). These geo‐demographic inequalities comprise sociospatial feedback loops. City centre luxury towers for young professionals counterpose outlying managed complexes for retirees, with people and capital flowing accordingly (Figure 7).

Joyce's complex sat amidst Wythenshawe's housing monoculture. She would have preferred something nearer amenities, but rents increased with proximity to shops, and she could not afford it. This became problematic when her dementia prevented long journeys. She regularly cancelled trips following a sleepless night and brain foggy morning. Lacking closer options, she would stay in her flat and wait for the next day, hoping to feel well enough to travel. Though lacking nearby amenities, Joyce's complex did have a bus stop. As with Paul's stop, it was a pole, which Joyce noted was inadequate for the needs of her co‐residents. She never mentioned it, but the pole seemed inadequate for her also.

The two buses that served her stop every 15 min provided Joyce with a lifeline following her driving licence revocation. She was diagnosed with vascular dementia during the second COVID‐19 lockdown and her licence had been withdrawn. When we met in 2023, Joyce's car remained in her carpark, unused for over two years. She relished bus travel but missed driving. At our first meeting, she told me that she had nearly completed the processes necessary to regain her licence. When we last spoke, another year later, I doubted that Joyce would drive again (legally, at least) given that her car still sat untouched and her health had noticeably deteriorated, instead remaining dependent on the buses, when her health allowed.

**FIGURE 8:**
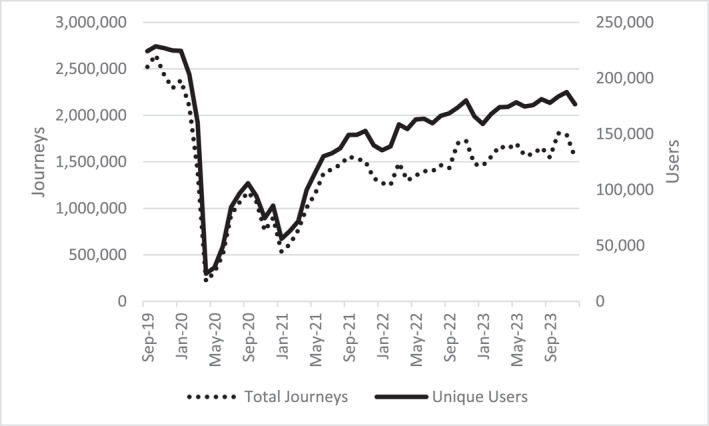
Older person's travel pass use across bus, train and tram. Data from TfGM via FOI request.

Joyce's health and mobility declines echo many studies highlighting deconditioning among older people during COVID‐19 lockdowns and especially people with dementia. A subset of this work attends to mobility, but largely focusses on physiological parameters of personal deconditioning (Felipe et al. 2023). Less explicated is the ecological deconditioning of mobilities, affecting infrastructures and places beyond passengers themselves. Recent data from Transport for Greater Manchester (TfGM) reveals that travel pass use by older people in late 2023 remained significantly below late 2019 use (Figure 8). When surveyed, 962 concessionary users attributed decreased postpandemic transport use to both personal deconditioning and worsening services (TFGM 2023), indicating the pandemic's overlapping capacities for human and infrastructural deconditioning.

Enmeshed deconditioning of passengers and infrastructures is also manifested spatially, again aligning with and intensifying underlying inequalities. In Figure 3, the pandemic's economic influence is evident in slight GDP growth declines in 2020. One can see the gentler impact on Manchester and Salford compared with other boroughs, exacerbating economic inequalities. Retail unit rental rates corroborate this story. By the end of 2021, of the 50 most populous British towns and cities, Manchester (3^rd^) boasted some of the highest retail unit tenancy rates per capita, whereas Stockport (46^th^) ranked among the highest vacancies (Davies 2021). Throughout IN‐CTU, high street retail was one of the most palpable manifestations of unequal spatial deconditioning. Most journeys were into centres for shopping. Joyce, like several others, measured a destination's quality via its shopping infrastructures and bemoaned recent closures. During every journey, she pointed out disused buildings, guiding me through a sort of bankruptcy safari:

Journey 1 There's a fair few shut down. Well, there was two travel agents here. Thomas Cook and the one opposite. But they closed down because of the covid and that

Journey 5 A load of pubs been pulled down. There's about 4, since covid, in Wythenshawe

**FIGURE 9:**
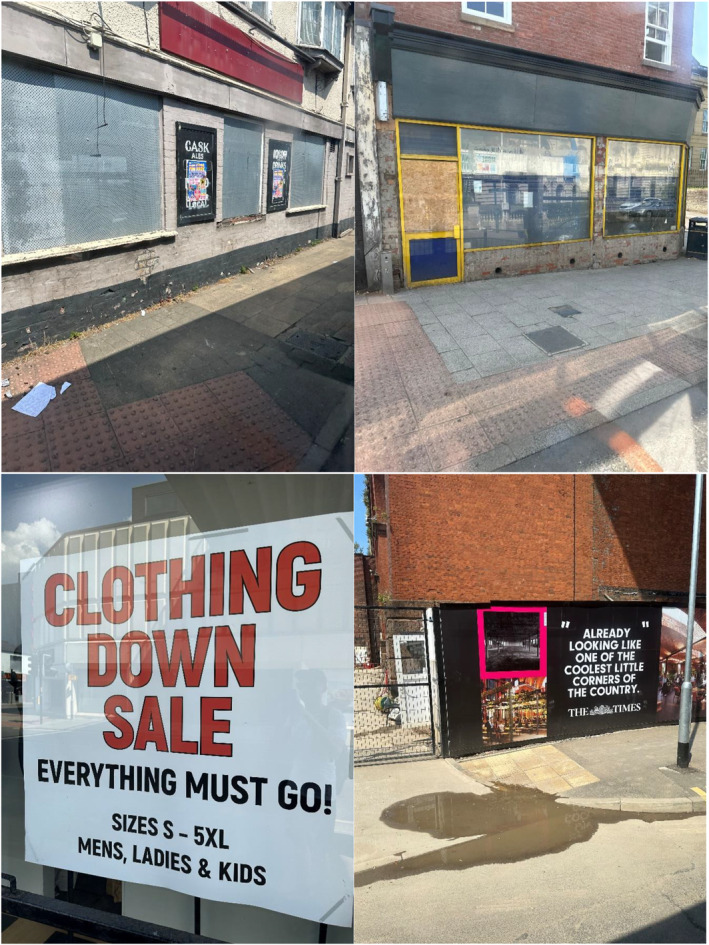
Joyce's photos of Stockport.

Joyce was fond of Stockport town centre, and I was impressed by its liveliness compared with other centres. Lots of people were shopping and various construction projects were underway. It was therefore interesting that many of Joyce's photos captured closed businesses and disused premises (Figure 9). This is perhaps because Stockport was one of the places where regional counter‐stories of ‘economic miracle’ and ‘ghost town’ collided. Adverts for investment opportunities in luxury developments sat next to vacant units. Her photos indicate how vanishing businesses weighed on Joyce's mind, even in comparably vibrant Stockport.

We recognise the disproportionalities of pandemic deconditioning and its ongoing legacies for older people, but this disproportionality extends to their places. Pandemic deconditioning, with its enmeshed spatial and temporal reverberations, was and is realised according to pre‐existing inequalities. Although a distant memory in some postcodes, in others it continues to worsen lives that can least afford worsening. As an example, the only walkable amenity near Joyce's complex was a shuttered social club down the road. It caught my eye as we disembarked a bus. The gates were padlocked and a litter picker was struggling to reach discarded cans through the railings:

Me Is it shut down, the social club?

Joyce Yeah, it is because, you know, it went when the covid

Me Oh, is that what finished it off?

Joyce Yeah. They used to have really good dos there

Me Are there no plans to open it up again?

Joyce I don't think so

In Manchester city centre, such a loss would be less likely, have less impact on community life and soon be replaced. In Wythenshawe, the abandoned social club was a tangible pandemic scar, a small local echo of seismic global events, materialised via, and thereby intensifying, regional economic inequalities. Each particular closure, however, seemingly inconsequential by itself, contributes to mutually reinforcing affective‐architectural decline, hastening capital flows away from struggling locales, endowing COVID‐19 with capacities to cause ideational and infrastructural harms akin to its capacities for biological harm.

## 6 Stanley: Sociospatial Lifecourses

Stanley was an archetypal resident of Didsbury, Manchester's most affluent suburb, which he described as ‘the Hampstead of Manchester’ when I noted the quality of his bus stop (large, covered, with seating and electronic schedules). His grand Victorian home belied a childhood in Moss Side, a historically deprived inner‐city area associated with gang violence. Stanley had benefitted from the grammar school system, before gaining local entry into the University of Manchester, and then winning a law scholarship in London. He had returned to Manchester for a successful career navigating the legal repercussions of deindustrialisation.

Stanley travelled into the city to visit libraries, museums and galleries. He planned to relinquish his driving licence imminently and was trying to use public transport more frequently in preparation. Our first journey was to Manchester Museum. The bus took us straight up Wilmslow and Oxford Roads, past Moss Side and the University of Manchester, tracing Stanley's history. He spent much of the journey relating his past, from working on his parent's hardware stall as a child through to establishing a law firm. That story was embedded in place. He recalled the building of much that we passed and what had preceded it. The mundane absences of those preceding spaces haunted and scaffolded Stanley's story in the manner depicted by Edensor's (2008) writing on Greater Manchester's provocative geographic absences. The route leant unique expression to Stanley's sociospatial lifecourse, a physical journey through his life's journey. Although we sat together, Stanley and I were seemingly travelling through different places vis‐à‐vis our personal spacetime relations.

Wilmslow/Oxford Road is claimed to be Europe's busiest bus corridor (though this is difficult to practically adjudicate), the university‐adjacent section boasting services every 30 s. Halfway between Didsbury and the universities lies Fallowfield, a suburb synonymous with students, with the University of Manchester's main accommodation housing several thousand students there. In 2021, 54.7% of the Fallowfield's population were students, compared with only 14.8% for Didsbury (MCC 2021). This has predictable age‐structuring consequences. Almost 1/3 of Fallowfield residents are aged 20–24, a remarkable concentration, whereas East Didsbury has Manchester's highest proportion of people aged 65+, at 14% (ONS 2021). Though geographically adjacent, Didsbury and Fallowfield are demographically antithetical.

Few forces shape the contemporary sociospatial character of Manchester as much as studentification. The University of Manchester, Manchester Metropolitan University, Royal Northern College of Music, University of Salford and University of Bolton have almost 120,000 students, with around 100,000 living in Greater Manchester. Studentification has long been criticised for urban displacement as buy‐to‐let investors acquire housing stock in disadvantaged neighbourhoods. The resulting densification of short‐term middle‐class youth migration has been criticised for intensifying sociospatial segregation. Latterly, a growing international investment market has expanded purpose‐built accommodation, including luxury high‐rises, with three of Manchester's 25 tallest buildings being student accommodation (Kenna and Murphy 2021).

The infrastructural consequences of studentification and sociospatial segregation materialise at Owen's Park bus stop in Fallowfield. Owen's Park is a long, covered bus stop, notorious for vast student queues. As our bus approached, I spotted the queue:

Me Are they all queuing for the bus?

Stanley Well, I think so, but I don't quite understand why they're round the corner

Me That's the longest bus queue I've ever seen

Stanley Yes, I think I'd agree with you on that. I mean, it's usually big. Must be the busiest bus

**FIGURE 10:**
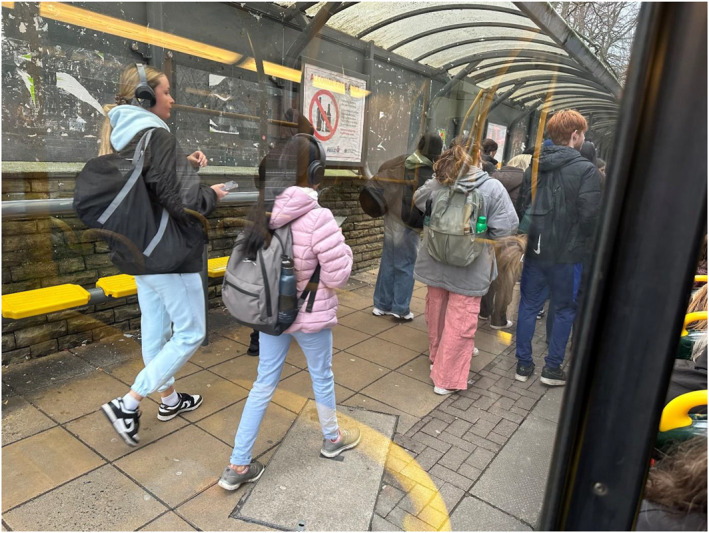
Students embarking at Owen's Park.

The bus stopped and the mass embarkation began (Figure 10). Sat side‐by‐side on the accessible seats at the front of the bus, Stanley and I were soon drowning in bums and bags. The second half of the journey was noisy, smelly and uncomfortable.

Our second trip was, again, to Manchester Museum (at the end of our previous journey, we had arrived to find it closed). When I entered Stanley's house, he was searching routes online, noting that he would need to get better at doing so before giving up driving. This time, we were taking the tram. The TfGM‐owned Metrolink is the UK's largest light rail system, with several lines fanning out across Greater Manchester from the city centre. The Didsbury section opened in 2014, and it was here that Stanley and I headed. We briefly waited in an open‐air concrete station, empty besides us, before embarking a tram. Riding along, I noted a map above us and was surprized by our circuitous route:

Me So, this tram route doesn't go directly up into the city. It loops round through Trafford and then it goes into the city?

Stanley Oh yeah

Me I've just noticed, I'm surprized that we're as far out as Trafford. We're doing a, sort of, loop?

Stanley Yeah. Yes, it was. But it's a lot better than going by bus, particularly avoiding the university centre

**FIGURE 11:**
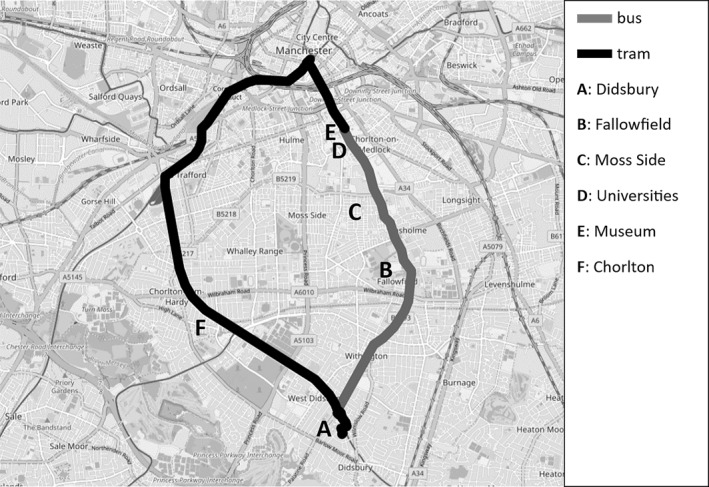
Stanley's routes.

I reflected on what was ‘better’ here. The bus ride had taken us north via Fallowfield and Moss Side. The tram looped around the southwest, through the up‐market suburb of Chorlton and the borough of Trafford (Figure 11). The tram infrastructure was evidently newer than the bus, and the tram itself far less crowded. ‘Better’ came at a price. My bus ride cost £2; my tram ride £3.80. Stanley benefitted from the older person's bus pass, providing free bus travel for people aged 60+. However, he paid a £10 surcharge to add tram travel to his pass. The pricier route offered correspondingly privileged views: quieter, cleaner streets, larger houses, more green space. Again, this entailed stark sociospatial segregation. 24.4% of Moss Side residents are white, compared to 80% of Chorlton's population. The average Moss Side income is £29,300 compared to Chorlton's £57,400 (Dubas‐Fisher and Timan 2023). Hence, Stanley's differing transport modes materially resonated with their sociospatial contexts.

After leaving Stanley at the museum, I reflected on his journeys as a confluence of sociospatial inequality across both his life course and that of the city itself. The Wilmslow/Oxford Road bus corridor offered an almost too‐perfect metaphor for his life course, a tale of personal mobility and prosperity amidst the wider neoliberal decimation of late‐20^th^ century northern England. Now, Stanley found himself dissuaded from that route by new forces of studentification and was able to use his accumulated advantage to pursue ‘better’ alternatives. At the same time, amidst national decay, Manchester prospered via studentification, just as Stanley benefitted from the enfranchisement of education decades earlier. For both Stanley and Manchester, higher education functioned as a medium of sociospatial mobility that intensified inequalities.

## 7 Barbara: Private Mobilities

Although Stanley exemplified implicit political materialities of regional mobilities, Barbara highlighted more explicit politics of local transport. A keen consumer of alternative news media, Barbara berated Greater Manchester's talismanic mayor, Andy Burnham, as an elite bureaucrat, removed from the travails of ordinary people:

They sit in an office. They probably live in Bramall or wherever. And they sit there and they’re telling us how we can get around on the buses. I mean, it’s like Andy Burnham, it’s alright for him, he’s chauffeur driven. He’s not got to stand in the cold for an hour, waiting for a bus.

She opposed pedestrianisation, bus lanes, 15‐min‐cities, and related urban planning fashions, dismissing them as anti‐freedom. Although at the extreme end of such sentiment, Barbara's advocacy of private car use was typical of participants. I was surprized by the commitment to automobility of people who had essentially been ejected from it. Like many others, Barbara had lost her driving licence when diagnosed with Alzheimer's disease and was the most frequent user of buses that I worked with. Hence, she seemed to advocate a mobility politics contradicting her own interests.

Barbara's counterintuitive position exemplifies the affective‐political life courses of mobility infrastructures. Older people came of age during the golden period of 20^th^ century automobility, whereby expanding private car ownership symbolised freedom and prosperity. These people can have little experience of public transport, rendering driving cessation hugely damaging, affectively and practically (Fletcher 2024c). Although cars are maligned in contemporary urbanism (i.e., from the perspectives of cognitively unimpaired university‐educated middle‐aged planners), their political and moral contingencies become more complex when we take seriously the distinct circumstances and histories of people's mobility and immobility (Cresswell 2010). Barbara had been a community nurse, and cherished her knowledge of local shortcuts and parking. She no longer drove, but hostility toward cars was nonetheless hostility toward her culture and her personally.

Although supporting private car use, Barbara was less keen on bus privatisation. IN‐CITU coincided with the Greater Manchester Combined Authority (GMCA) instituting its Bee Network, the first re‐municipalisation of buses in the UK since the 1980s. The *Transport Act 1985* privatised and deregulated previously publicly owned British bus services. The *Bus Services Act 2017* allowed mayoral combined authorities to partially re‐regulate via franchising, which Andy Burnham proposed GMCA do. This proved contentious. Rotala, the conglomerate who own local bus operator Diamond, lobbied against the Bee Network. They even took GMCA to court, losing the trial and subsequent appeal. The first Bee Network services began in September 2023, including Barbara's usual routes, which until that time were operated by Diamond.

**FIGURE 12:**
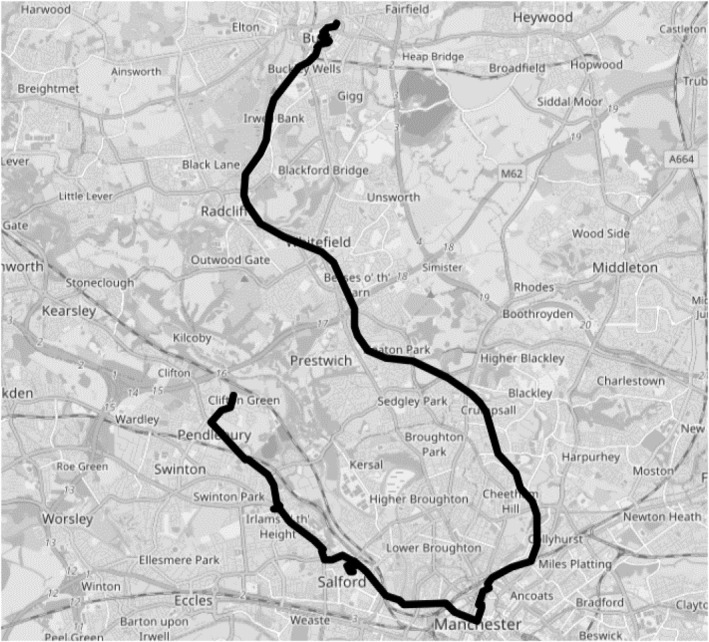
Clifton to Bury.

Barbara disliked Diamond. Her complaints started immediately during our first journey from her home on Salford's outskirts to Bury market. Her first problem was the route itself. As with many of Greater Manchester's public transport journeys, it required an inner‐city connection, which entailed first travelling away from Bury for 40 min (Figure 12). Barbara complained that bus services that had once connected outer towns were being cancelled because they were unprofitable. Several disused bus stops on Barbara's estate evidenced this unprofitability. These buses had stopped winding through the estate, in favour of the main road in and out of Manchester. Now Barbara had to walk 10 minutes, passing the discontinued stop at the end of her street. Covered and seated, this abandoned stop was nicer than the poles still functioning as stops elsewhere.

Once at the in‐use stop, Barbara complained about bus disrepair, particularly dirtiness, which she again attributed to profiteering companies:

They never get cleaned, do they? They don’t pay the price now for people to put any pride into anything, do they? I mean, if they’re cleaning buses, they’re probably on minimum wage. And they’re expected to do 200 buses a night or something like that. They’ll have a set target that they’ve got to do and if they don’t do it then they just sack them, don’t they?

As our bus arrived, Barbara mentioned that a friend had told her that this service was terminating at a new stop. It used to go directly to Victoria station, where we would be catching our connecting tram to Bury, but it now terminated a few streets away, outside M&S. Embarking the bus, Barbara asked the driver and he confirmed. Barbara received all her travel updates via word‐of‐mouth and drivers, as did most participants. I researched the service change on Diamond's website, which explained that city centre traffic was causing regular standstills between M&S and Victoria. I explained this to Barbara, who protested:

That’s not right though, is it? Well, they don’t run it for the people, do they? I’m hoping when the Bee buses come, they’ll be running them for the people instead of the company.

**FIGURE 13:**
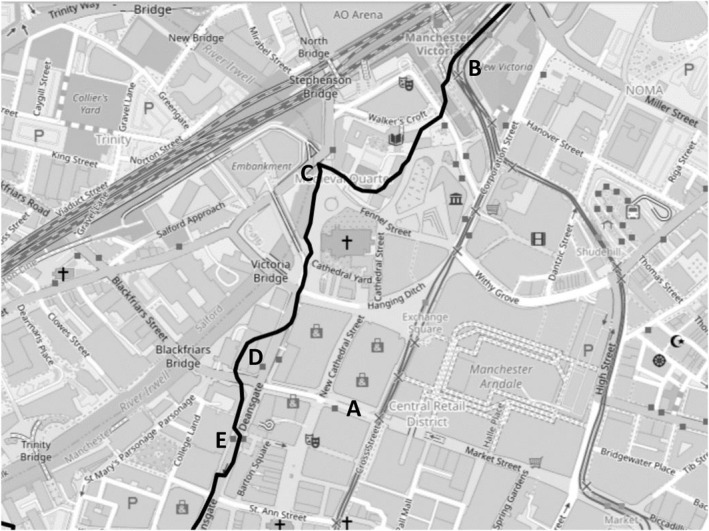
Victoria‐diamond (dis)connection.

Diamond's route change did not hinder our outbound journey. We completed the connection on foot and caught a tram to Bury. The return journey was different. Barbara assumed that the return bus would depart from M&S (Figure 13A), but the driver of the outbound bus refuted this. Back at Victoria station (Figure 13B), Barbara used the Diamond app to find the bus stop, ND, and I cross‐checked it on Google maps. We set off on foot again, around the cathedral and down to what we thought was ND, ending up in a construction site (Figure 13C). We turned back and walked down the main road until we arrived at ND, another pole with a sign attached, thankfully including our bus number (Figure 13D). We waited; nothing came. We eventually noticed the road was blocked further down due to an adjacent demolition. Disorientated and disheartened, Barbara reflected on her situation:

I’m not going come to Manchester because there’s no way I would be able to find how I can get the bus. So, it’s really spoilt my way of doing this. Just dropping that bus from two stops has really put my plans out of action. It’s pushed me out. Really pushed me out. Because I’m not confident. I don’t want to get lost. You know what I mean, because if I once got lost, I’d never go out again. And that would really bring me down, I think. So, I just don’t know. Coming out with you today has made me think that things have changed big.

Shortly thereafter, a not‐in‐service bus pulled up with a driver Barbara recognised. He explained the stop was discontinued, but the signs and app had not been updated. He directed us to another stop (Figure 13E), where we finally caught our bus (see Fletcher 2024d for in‐depth analysis of micro‐interactions and relationships in IN‐CITU).

The material ramifications of global, national and local political economy rippled through Barbara's (im)mobility. Profitability demanded perpetual route evolution in response to the city's re‐materialisation, itself driven by international investors fuelling frenetic construction, as diversions, closures and jams imperilled operators' dividends. Of the left‐behind infrastructures scarring the region, some were more obviously disused than others, and even the app failed to keep pace. Lost in a demolition dust cloud, I found myself questioning for whom this place was ‘friendly’. I reflected on how difficult this experience had been for me as an able young man doing research, and how much worse it would have been had I been an older woman with cognitive impairment trying to live my everyday life. Our journey revealed amplifying and reifying materialisations of profitability and disability, eroding the city's navigability, and indeed habitability, for people with cognitive impairments, incentivising them to self‐isolate and risk further disablement, while refining its capacities for value extraction, with consequent passenger disengagement feeding back into the financial unviability of services.

## 8 Discussion

The four cases described in detail, combined with the briefer examples from other participants, are as idiosyncratic as the all‐ageing metropolis itself. This warrants celebration as a means of resisting dementia‐friendly initiatives that seek to homogenise dementia and place as fixed entities. The disciplinary study of later life has long suffered a tendency toward determinist demographic thought styles, wherein vast categories of person are collated as a means of making questionable—and often politically motivated—claims about the nature of ageing, later life and disability (Fletcher 2021a, 2021b). This resonates with interventionist dispositions that target dedicated technical fixes to problems that, once narrowly defined, are set to be narrowly solved. The stories from IN‐CITU trouble such categorisation, narrowing and intervention by unequivocally demarcating later life cognitive decline as an everything‐problem. By this I mean that the quotidian difficulties experienced by passengers with dementia in real life are irreducible to root causes and predictable effects. They can simultaneously be as beholden to a single metal pole as a Middle Eastern investment fund, all under the press of a specific but slippery mass of circumstances. Although we quite understandably seek narrowed‐down dementia problems and targeted dementia interventions, it is important to take seriously the everythingness of everything‐problems such as ageing and cognition. As the above accounts underscore, that everythingness is integral to how they exist in the real world, and I would argue that this requires our attempts at knowledge‐making to be correspondingly sensitive to everything, hence the nonrepresentational and multimethod approach in this study (though inevitably much will have still been missed).

Nevertheless (yet also in the same vein), beneath their particularities, the above stories collectively exemplify the challenges generated by a material politics of urban ageing that constrains the conduciveness of places to good lives with dementia. This is an observation that is readily extendable to the experiences of all the passengers who contributed to IN‐CITU. We have long recognised the ‘shrinking world’ as a core negative trajectory of many people's experiences of living with dementia, whereby the range of places that a person spends time gradually decreases, often leading to lives lived exclusively between a handful of rooms in a house (Duggan et al. 2008). Such scholarship has noted the importance of accessibility and familiarity to facilitating continued multi‐place engagement for people with dementia but has largely taken for granted notions of accessibility and familiarity as intrinsic architectural and neurodegenerative contingencies respectively, with little consideration of the potential for places—let alone spacetimematterings (Gallistl and Wanka 2023)—to change far faster and more profoundly than a person's cognitive abilities (R. Ward et al. 2022b). This is particularly true of change driven by political economic forces that directly or indirectly exclude older people and those with cognitive disabilities, from the digitalisation of information infrastructures to the landlordisation of regional housing stock. An interesting consequence of these meta‐transformatory forces vis‐à‐vis ageing and place was my overriding sense that, despite my travel companions having lived in the city for many decades, compared with my 2 years, I was in many ways more of a conventional ‘local’ than they were, comfortably situated in spaces cultivated by imaginaries of mobile young professionals that chimed with my life at the same time that they alienated others. Too little work on dementia and place acknowledges and grapples with these ideological contingencies and the corresponding transience of the ecologies with which people age, think and experience dementia.

We might anticipate that the journeys of IN‐CITU participants would be made difficult by cognitive impairment, yet their mobilities were at least as constrained by the sociospatial and affective‐architectural consequences of entangled global, national and regional political economies, from privatisation to studentification. However, this paper is not an anti‐gentrification or anti‐investment diatribe. It would be easy (but wrong), to weave a tale of politicians and developers chasing wealthiness at the expense of friendliness. That is, not the reality of IN‐CITU participants who generally welcomed regeneration. Judy, who lived in Salford's inner‐city ward of Trinity, recalled the area's deprivation and criminality during the 1980/90s. She now bragged about living in (what she called) ‘Trinity Village’, replete with a vegan cafe, tapas bar and combined bakery‐gallery, not that she frequented them. Participants valued Manchester's revitalisation, even when they were excluded from it, even by it. Such perspectives, from long‐term residents, are vital to understanding the contradictions of urban regeneration (Buffel and Phillipson 2019). They reveal how, in practice, the complex political materialisations that are often labelled as ‘gentrification’ can be contradictory, simultaneously improving and worsening peoples' lives under the influence of overlapping progressive and extractive rationales. These findings echo those of Barron's (2019) nonrepresentational go‐along research with older people in revealing the complicated relationship of lifecourse with chronological time. Though often misrepresented as analogous, lifecourse is bound to geographical stasis and transformation in ways that transcend and even contradict stage‐based chronological time.

My broader aim here is to reveal, in real‐life detail, how the vitalities of place—as articulated by new materialism—and their capacities to materialise (dis)abilities and (im)mobilities—the core focus of ‘friendly’ scholarships and initiatives—are acutely political and economic, simultaneously cutting across global and personal phenomena. Pursuing a new materialist approach, Fox and Powell (2021) have interjected in the ‘lacuna’ between historical materialist and cultural‐turn sociologies of health inequity, emphasising the role of ‘a thousand tiny dis/advantages’ that characterise human‐material relations. Such work makes a valuable contribution, but a critical new materialism must not abandon direct discussion of larger scale political economic concerns. The journeys discussed above impress upon us the multitudinous vitalities of place and its capacities to materialise disability at scale. Skyscrapers bloom, viruses scar highstreets, studentification overwhelms infrastructures and private profits undermine personal wayfinding. Rather than a neutral story of matter's capacities to matter, the ageing metropolis is a political materialisation, animated by entangled global, national and local political economies and competing interests, all inscribed into the buildings we pass and the accents we hear as we walk down a street. Andrews, Griffin and Phoenix (2024) describe these combined sociospatial interactions as ‘onflow’—the stream of experience that ultimately generates our consciousness. A critical ‘all‐ageing’ approach to disability can attune to these influences as the flows through which disability is materialised, and hence what types of people are more or less able to be in what types of places, under the material press not only of time but also of specific political economic machinations.

Ageing‐in‐place scholarships could gain from this critical new materialist disposition. Echoing ‘friendly’ agendas, the recent focus on social inequalities in sociological work on ageing and disability has been typified by the simplistic stratification of outcomes by various social groupings, and corresponding conclusions that X group should pursue X behaviour to improve their outcomes (Fletcher 2024a). I would argue that it is difficult to reconcile that approach with IN‐CITU participants' experiences. There is little that Barbara could do to mitigate Diamond's shapeshifting services, nor Joyce to reverse pandemic closures. Approaches that reify inequalities to social categories (e.g., old and disabled) and impute some essential coherence across, and thereby despite, different political‐material locations risk obscuring—and perhaps perpetuating—the dynamic forms of exclusion that emanate in urban ecologies. Misleading simplifications of both dementia and place inform contemporary policy in this manner (R. Ward et al. 2018). Dementia functions as such an obscuring category, automatically conjuring assumptions about what kinds of problems those people face (e.g., forgetting things) and thereby focussing our attentions on corresponding fixes (e.g., signage) at the expense of problems that feel intuitively irrelevant to dementia (e.g., studentification). In practice, life with dementia and life with place are co‐constituted by powerful political economic considerations, generating circumstances within which installing dementia‐friendly signage can seem impotent (R. Ward et al. 2021). Social analyses of ageing and disability, as they are lived sociospatially, demand that we re‐evaluate, if not re‐form, how marginalised people come to matter (or not) in the metropolis as a political material amalgam.

Andrews and Read (2024) ultimately criticise the fledgling new materialism of ageing and place, including work on dementia, as remaining somewhat tied to the human as *the* ageing subject. This paper has sought to respond to that critique by centring (1) Manchester and (2) political economy as some of the core ageing subjects under examination. As ‘an emergent and relational capacity produced and reproduced in everyday material interactions’ (Alldred and Fox 2019), how people matter is shaped by relations with the political materialities of place (e.g., property ownership and transport proximity). The capacities of those relations to materialise how a person matters owe their power to social, political and economic relations. The mattering of people via material relations is vitalised by the political motivations and activities of various stakeholders. It is made potent by overlapping investor, politician, developer, student, business, resident and passenger power struggles, evoking broader classist, ageist and ableist notions of urban desirability and decrepitude. New materialist scholarship can deconstruct corresponding political materialisations of infrastructures, for example, how a metal pole comes to function as a bus stop in a cold, wet, rich city, whose interests are served by the pole and who it disables. The political materialisation of these everyday places in line with powerful interests (e.g., Abu Dhabi royalty and university management) and ideologies (e.g., financialisation and austerity) risks intensifying existential inequalities (Neves et al. 2023). A pole, an app, a diversion—they aggregate to materialise the distribution of who matters, and how much, along existing axes of inequality.

## Author Contributions

**James Rupert Fletcher:** conceptualization, data curation, formal analysis, funding acquisition, investigation, methodology, project administration, resources, software, validation, visualization, writing–original draft preparation, writing–review and editing.

## Conflicts of Interest

The author declares no conflicts of interest.

## Data Availability

The data that support the findings of this study are available on request from the corresponding author. The data are not publicly available due to privacy or ethical restrictions.

## Endnotes

^1^
Dependency ratios denote the number of 16–65‐year‐olds for every person aged 66+.
